# Supplementary material for: A comprehensive HPV-STI NGS assay for detection of 29 HPV types and 14 non-HPV sexually transmitted infections
Source: Infect Agent Cancer. 2022 Mar 21;17:9. doi: 10.1186/s13027-022-00420-8 (PMC8935747; doi:10.1186/s13027-022-00420-8)
Supplement: Supplementary file 1 — Additional file 1. Comparison of Illumina sequencing platforms and sequencing kits for HPV-STI analysis. [file 13027_2022_420_MOESM1_ESM.docx]

Supplementary Table S1. Comparison of Illumina sequencing platforms and their respective sequencing kits for HPV-STI analysis

| Illumina Platforms | Illumina Seq. Kit | Read Length | Output | Reads/Run | Samples |
| --- | --- | --- | --- | --- | --- |
| iSeq 100 | iSeq 2 x 150 | 2 x 150 | 1.2 Gb | 4 M | 192 |
| MiniSeq | Mid-Output Kit | 2 x 150 | 2.4 Gb | 8 M | 384 |
| MiniSeq | High-Output Kit | 2 x 150 | 7.5 Gb | 25 M | 1,200 |
| MiSeq | MiSeq v2 Nano | 2 x 150 | 300 Mb | 1 M | 48 |
| MiSeq | MiSeq v2 Micro | 2 x 150 | 1.2 Gb | 4 M | 192 |
| MiSeq | MiSeq v2 | 2 x 150 | 4.5-5.1 Gb | 12-15 M | 576-720 |
| NextSeq | Mid-Output Kit | 2 x 150 | 32.5–39 Gb | 130 M | 6,240 |
| NextSeq | High-Output Kit | 2 x 150 | 100–120 Gb | 400 M | >10,000 |
